# Evidence for the Accumulation of Nonsynonymous Mutations and Favorable Pleiotropic Alleles During Wheat Breeding

**DOI:** 10.1534/g3.120.401269

**Published:** 2020-09-08

**Authors:** Elie Raherison, Mohammad Mahdi Majidi, Roos Goessen, Nia Hughes, Richard Cuthbert, Ron Knox, Lewis Lukens

**Affiliations:** * Department of Plant Agriculture, University of Guelph, Ontario, Canada; † Department of Agronomy and Plant Breeding, College of Agriculture, Isfahan University of Technology, Isfahan, 84156-83111, Iran; ‡ Swift Current Research and Development Centre, Agriculture and Agrifood Canada, Swift Current, Saskatchewan

**Keywords:** breeding, identity by descent, de novo mutation, pleiotropy, polyploidy

## Abstract

Plant breeding leads to the genetic improvement of target traits by selecting a small number of genotypes from among typically large numbers of candidate genotypes after careful evaluation. In this study, we first investigated how mutations at conserved nucleotide sites normally viewed as deleterious, such as nonsynonymous sites, accumulated in a wheat, *Triticum aestivum*, breeding lineage. By comparing a 150 year old ancestral and modern cultivar, we found recent nucleotide polymorphisms altered amino acids and occurred within conserved genes at frequencies expected in the absence of purifying selection. Mutations that are deleterious in other contexts likely had very small or no effects on target traits within the breeding lineage. Second, we investigated if breeders selected alleles with favorable effects on some traits and unfavorable effects on others and used different alleles to compensate for the latter. An analysis of a segregating population derived from the ancestral and modern parents provided one example of this phenomenon. The recent cultivar contains the *Rht-B1b* green revolution semi-dwarfing allele and compensatory alleles that reduce its negative effects. However, improvements in traits other than plant height were due to pleiotropic loci with favorable effects on traits and to favorable loci with no detectable pleiotropic effects. Wheat breeding appears to tolerate mutations at conserved nucleotide sites and to only select for alleles with both favorable and unfavorable effects on traits in exceptional situations.

Modern plant breeding, characterized by controlled crosses and progeny evaluations, is expected to have distinct effects on nucleotide variation and traits’ genetic architectures relative to plant improvement as was practiced during domestication. Crop breeding methods ensure the selection of genotypes with high values for traits of interest in target environments. Many new cultivars in crops such as barley, wheat, bean, and soybean are developed from a biparental cross of elite lines. From this F1 cross, often hundreds of individual F2 seed give rise to distinct inbred families. These families are evaluated and almost always eliminated. Thousands of lines may be evaluated in head rows (F5), 100s in a preliminary yield trial (F6), 10s in an advanced yield trial (F7), and a handful in registration trials (Figure 1A). As lines become closer to becoming cultivars, the effort in obtaining accurate genotypic estimates grows. Breeding lines are evaluated across several years and locations in formal experiments that minimize and account for environmental effects.

**Figure 1:**
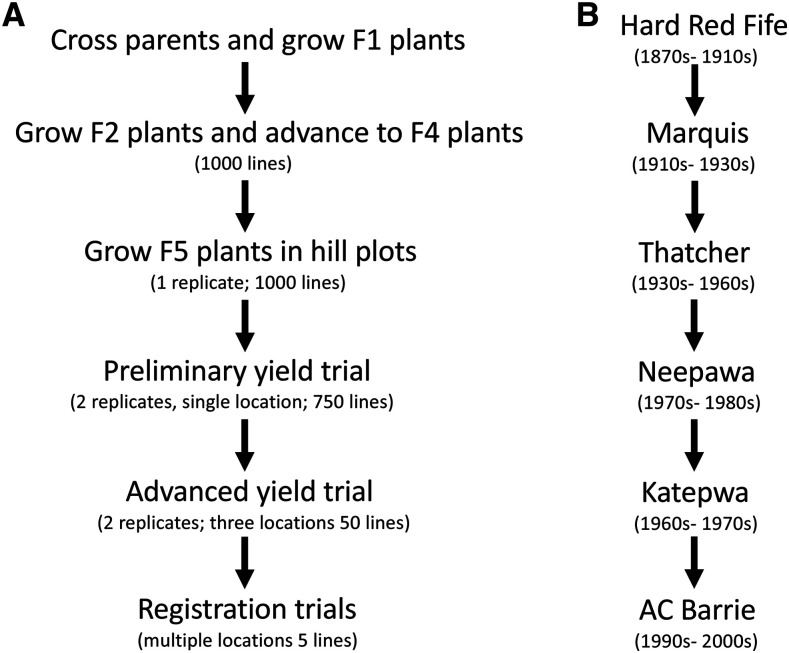
A depiction of a plant breeding cycle and the lineage of major Canadian hard red spring cultivars. (A) A graphical representation of a breeding cycle starting with a cross and ending with a registered cultivar. The historical breeding processes that generated Stettler and its progenitors would differ from this representation. For example, backcross breeding was used to introduce disease resistance alleles into cultivars and family numbers varied. Nonetheless, the diagram has the key attributes of cultivar development: rigorous evaluation of hundreds of lines and selection of a very small number. (B) The most widely grown hard red spring cultivars in Canadian prairies over different eras are shown (McCallum and DePauw 2008). Cultivars’ pedigrees are complex, but each dominant cultivar is derived from a previous cultivar. For example, Marquis was derived from Hard Red Fife; Thatcher from Marquis; and Neepawa from Thatcher. Prodigy and Superb, the parents of Stettler arose from the same germplasm and were grown on a significant area in the early 2000s. A full pedigree is provided in McCallum and DePauw 2008.

Because of selection for high value genotypes, *de novo* deleterious alleles that arise during breeding are unlikely to accumulate in cultivars. Non-ancestral, nonsynonymous SNPs, especially at conserved sites, can be assumed to have a high proportion of deleterious effects (Keightley and Lynch 2003; Eyre-Walker 2006). Several studies have reported that the frequencies of mildly deleterious alleles segregating in wild populations rise during domestication and landrace improvement. Deleterious alleles in domestication lineages are thought to amplify due to small population sizes, dominance of favorable alleles, and linkage to positive alleles. Genetic drift and dominance can have small effects on breeding populations. Selected genotypes are largely homozygous and produce close to genetically identical progeny (Figure 1A). In addition, linkage between a deleterious allele and a positive allele is unlikely. A line in which a deleterious mutation arises would need to out-perform many full-sibling derived lines in which the deleterious mutation did not occur (Figure 1A). Gaut *et al.* (Gaut *et al.* 2015) suggested that missense variants will be more effectively reduced in elite cultivars compared to landraces because of the strong selection for yield in elite cultivars. The frequency of missense variants relative to synonymous variants was relatively low in sunflower cultivars relative to landraces (Renaut and Rieseberg 2015), although the authors attributed this pattern to the introgression of wild alleles into cultivated germplasm.

Many domestication alleles’ effects are independent of other loci indicating that selection favored genes with stable effects on traits (Doust *et al.* 2014). Early agriculturalists would be presumably more likely to select genotypes that always expressed a trait instead of genotypes that expressed a trait intermittently depending on genetic background. In plant breeding, stable positive effects are also desired, but pleiotropic alleles with favorable effects on some traits and unfavorable effects on others seem more likely to be utilized. Breeders may focus on a key gene with positive effects on a desired trait and compensate for its negative effects with modifying loci. Genotypes may pass through a phase of low fitness while the modifying alleles are identified. There are a number of examples of compensation for genes that have positive effects on some traits and negative effects on others. Enhanced kernel lysine content in maize, which improves feed quality, has a number of negative effects on other traits that can in part be altered by modifying alleles (Wessel-Beaver and Lambert 1982). In sweet potato, beta carotene levels and yields are negatively correlated, and modifying loci have generated lines with high values for both (Afuape *et al.* 2019). Quantitative trait loci (QTL) mapping can identify loci that contribute to compensation. For example, in rice, Wang *et al.* (Wang *et al.* 2012) showed an allele at a single locus, Ghd7/qHD7, compensated for an allele at a second locus Ghd8/qHD8. The 93-11 allele of Ghd8/qHD8 greatly enhances yield, a desirable effect, but contributes to late flowering, an undesirable effect. The 93-11 allele has a high yield but a small effect on flowering time when coupled with a Zhenshan97 allele of Ghd7/qHD7.

Here, we investigate the accumulation both of missense and other putatively deleterious mutations and of negatively pleiotropic genes and compensatory alleles during wheat breeding. Red Fife, introduced in 1860, produced very high-quality grain for breadmaking and is a direct ancestor of Stettler, released 150 years later (Figure 1 B) (McCallum and DePauw 2008). To understand the nature of mutations that have accumulated during plant breeding, we identify Red Fife and Stettler chromosomal sequences that very recently diverged. The nature of nucleotide differences within these chromosomal regions can determine if selection purged mutations that are normally classified as deleterious. Wheat has over 100,000 genes, providing a large target for these infrequent mutations (Appels *et al.* 2018). To assay if pleiotropic alleles and compensatory loci distinguish Red Fife and Stettler, we identify genotypic correlations for agronomic traits and identify QTL in a population derived from the two parents. Trait correlations would indicate the presence of pleiotropic loci. Stettler QTL alleles with favorable effects on one trait and unfavorable effects on other traits indicate a negatively pleiotropic locus, and Stettler QTL alleles that counteract negative effects would identify compensatory alleles. Stettler contains the green revolution semi-dwarfing gene allele *Rht-B1b* that may have had unfavorable effects that were compensated by other loci.

We find mutations that accumulated during wheat breeding frequently caused missense mutations and occurred in conserved genes. The mutations’ effects on proteins were indistinguishable from random mutations’ effects suggesting the absence of purifying selection. It seems likely that normally deleterious alleles have accumulated during plant breeding with low or no cost to crop plant performance. Nonetheless, these new alleles may constrain genetic performance in environments and genetic backgrounds other than those targeted by past plant breeders. The genetic analysis showed that all traits with the exception of plant height had favorable genetic correlations. Favorable plant heights were correlated with unfavorable values for other traits. The *Rht-B1* locus was largely responsible for plant height variation in the Red Fife x Stettler population, and Stettler alleles at other loci counteracted *Rht-B1b*’s negative effects on seed weight. Modern wheat breeders, like early agriculturalists, infrequently selected for loci with unfavorable effects that required genetic compensation.

## Materials and Methods

### Plant materials and trait measurements

A set of 226 doubled haploid (DH) lines were each derived using microspore culture of F1 plants from a cross between the Red Fife and Stettler hard-red spring wheat cultivars. Stettler was released in 2008 and has high yield potential, protein content, lodging resistance, orange wheat blossom midge resistance, and rust resistance (DePauw *et al.* 2009). Red Fife is highly inbred, and Stettler is a double haploid. For field performance traits, the 226 DH lines, Red Fife, and Stettler were evaluated for eight traits over two locations with two replicates per location. Raw data are available in Table S1. We used linear models to estimate genotypic means and trait heritabilities. Supplementary Materials and Methods provides more information on trait measurements and statistical analyses. Genetic correlations within the population were due to pleiotropy, in which allelic differences in one gene affected phenotypic values of multiple traits, or due to linkage disequilibrium, in which two or more polymorphic linked genes affected different traits. Genetically distinguishing pleiotropy from linkage is a major challenge, and the term “pleiotropic locus” refers to both scenarios.

### RNA isolation, sequencing, read mapping, transcript abundance estimation, SNP identification, and SNP calling

RNASeq data were used to quantify genes’ transcript abundances in Red Fife and Stettler and to identify SNPs between them. Details on plant growth, RNA isolation, library preparation, and library sequencing are given in Supplementary Materials and Methods. The 2^nd^ leaves from the bases of 12 day-old growth room plants were harvested. Red Fife and Stettler were each represented by four samples. Leaves from plants grown across three replicates in time were pooled into a single sample. Each sample generated between 20-60 million 100-125nt paired end sequence reads that were aligned to the Chinese Spring reference genome (v1.0) with STAR v 2.5.2b (Dobin *et al.* 2013) (Table S2).

**Figure 2:**
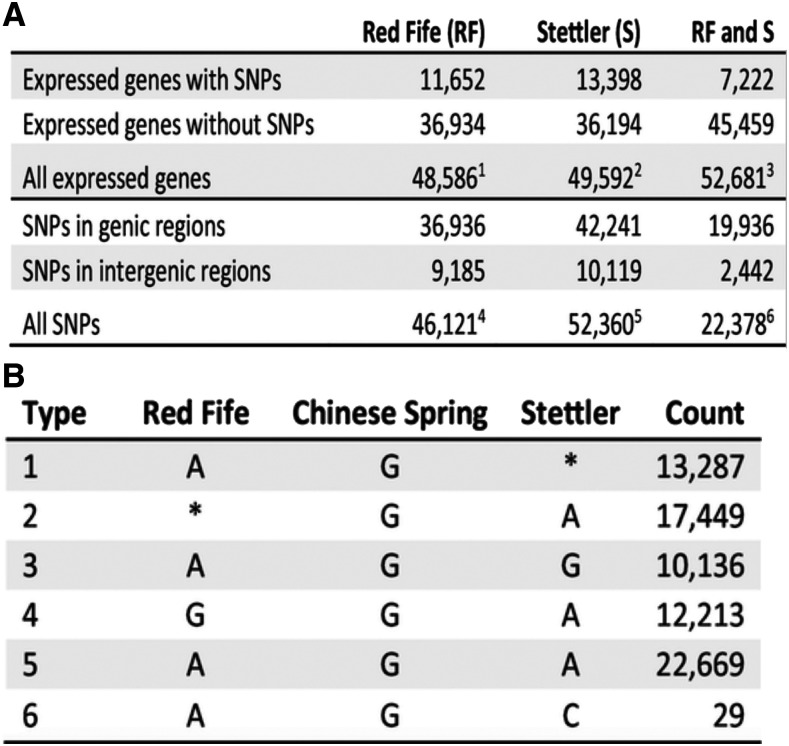
RNA-Seq detected SNP counts between cultivars. (A) Numbers and attributes of putative SNPs within expressed genes and genic and intergenic regions. ^1^Total number of genes expressed in Red Fife (RF); ^2^Total number of genes expressed in Stettler (S); ^3^Total number of genes expressed in RF or S; ^4^Total number of SNPs between RF and Chinese Spring wheat (CS); ^5^Total number of SNPs between S and CS; ^6^Total number of SNP markers or SNPs between RF and S. (B) Numbers of putative SNPs that are polymorphic between RF and CS, S and CS, and RF and S. There are six types of SNPs. These include SNPs between RF and CS (type 1, 3, 5 and 6), between S and CS (type 2, 4, 5 and 6), and between RF and S (type 3, 4 and 6); * indicates that a nucleotide was not called for a position in alignments to the CS reference genome.

Gene transcript abundances in Red Fife and Stettler were estimated using DeSeq2 (Love *et al.* 2014). Detailed methods are provided in Supplementary Materials and Methods. Alignments of Red Fife and Stettler reads detected close to 1/2 of the annotated genes. Of the 110,790 annotated genes, 44.8% were expressed in Stettler, and 43.9% were expressed in Red Fife (Figure 2A). Mean transcript abundances were calculated across the eight samples and are given in Table S3.

While aligning reads to templates generated by incomplete and/or incorrect *de novo* transcript assemblies or NCBI unigene models may mis-identify SNPs between homeologous loci as SNPs between homologous loci (Ramirez-Gonzalez *et al.* 2015), mapping reads from different genotypes’ genomic DNA to high quality reference sequence has identified many biallelic nucleotide positions (Lorenc *et al.* 2012; Jordan *et al.* 2015; Rimbert *et al.* 2018). SNPs between Red Fife and Stettler and Chinese Spring were called using SAMtools v1.3.1, and then we identified SNPs between Red Fife and Stettler. To be called, a SNP required detection in at least three of the four parental samples as described in Supplementary Materials. 46,121 SNPs were identified between Red Fife and Chinese Spring, and 52,360 SNPs were identified between Stettler and Chinese Spring (Figure 2A, Figure 2B). Some SNP sites, for example 2.9% of the SNP sites between Chinese Spring and Red Fife (1,335 of the 46,121) had different nucleotide calls at sites, possibly indicating alignment of homeologous or paralogous sequences to a single reference genome position. Nonetheless, at these sites, reads with one nucleotide greatly predominated over reads with the other (Figure S1). 22,378 SNPs were identified between Red Fife and Stettler with 19,936 within genes (Figure 2B; Table S3). Chinese Spring is known to be genetically distant from modern wheat breeding lines (Thind *et al.* 2018). Additional details on SNP calling are provided in Supplementary Materials.

### Evaluation of SNP effects and identification of putative shared chromosomal regions and triplicated genes

An objective of this work was to understand the attributes of nucleotides that accumulated during plant breeding. To do this, we identified nucleotide polymorphisms that putatively arose since modern plant breeding was initiated. We distinguished new polymorphisms from old polymorphisms by the genomic regions in which they occurred. Red Fife/ Stettler polymorphisms in recently diverged chromosomal segments were considered new. Polymorphisms in unrelated chromosomal segments were considered old. Estimated substitution rates in maize, a related grass species, are 1.63 × 10^−8^ substitutions per site per year (Jiao *et al.* 2012) and between 2.9 × 10^−8^ to 3.3 × 10^−8^ substitutions per site per year (Clark *et al.* 2005). Current Red Fife, the cultivar derived from selfing the ancestral Red Fife, and Stettler have diverged for over 300 years, so we expected 4.9/ 9.3 mutations per million bp in recently diverged chromosomal sequences. We expected Red Fife and Stettler to have long, shared chromosomal regions because of their relatedness (Figure 1B), and we defined regions of 200 contiguously expressed genes with 2 or fewer SNPs as closely related. The expected number of SNPs per 200 genes was 1.42/2.70 assuming an average wheat transcript length of 1,453 bp (Ling *et al.* 2018). New mutations could not be detected in small chromosomal regions. For example, a region of 50 genes with an expected length of 72,650 bp would not be expected to have a single SNP given the highest mutation rate. Regions were defined as related or unrelated using a sliding window starting at the top of a pseudochromosome and defining new, 200-gene intervals for every five genes until the end of the pseudochromosome was reached. Code for this analysis is in Supplementary Methods.

To predict the effects of SNPs between coding sequences in related regions and unrelated regions, we used snpEff v4.3T (Cingolani *et al.* 2012). Within coding sequences, four classes of SNPs were present with predicted effects on protein function given in parentheses: “stop gained” (high), “missense variant” (moderate), “missense variant and splice_region_variant” (moderate), and “synonymous variant” (low). The “missense” classes were pooled and termed nonsynonymous variants. To compare the effects of random, coding sequence SNPs with the effects of observed SNPs, we extracted the longest sequence from each expressed gene and altered 93,993 nucleotides to another nucleotide using the frequencies observed from SNPs detected in the related chromosomal regions. Code for this analysis is given in Supplemental Materials and Methods.

We used SynMap (Haug-Baltzell *et al.* 2017) to classify genes based on their presence/absence in the three wheat subgenomes. SynMap was downloaded from the CoGe web platform (https://genomevolution.org). We used default values for the software. A gene is considered “non-triplicate” if there are not three syntenic copies of the gene; “unique triplicate” if there are only three syntenic copies; and “non-unique triplicate” if there are three syntenic genes and one or more additional genes elsewhere in the genome. Genes’ triplication statuses are given in Table S3. Details are reported in Supplemental Materials and Methods.

### Genetic map construction and QTL identification

To map QTL for agronomic traits, we used SNPs identified between Red Fife and Stettler to genotype the DH population. RNA-Seq was performed on 154 DHs that were randomly selected from the 226 field grown lines. RNA from each DH line was represented by one tissue sample pooled from samples collected over three timepoints. RNA isolation, library preparation, sequencing, and read alignment were done as with the parental lines.

The 22,378 Red Fife and Stettler SNPs were called across the DH population (Table S4). 15,497 passed detection and segregation distortion filters and were placed into 25 linkage groups that corresponded to pseudo-chromosomes or pseudo-chromosome regions (Tables S5 and S6, Figure S2) using R/ASMap (Taylor and Butler 2017). The 4,010 cM map had 1,503 distinct genetic map positions (Figure S2), although for some loci only one marker genetically linked flanking markers (Table S5). 45 SNPs (0.3%) mapped to linkage groups that differed from their pseudochromosome expectations (Table S5). Most of these 45 SNPs genetically mapped to chromosomes homeologous to the chromosomes of their physical map (Table S5). Thus, genome assembly errors whereby sequence from one chromosome was incorrectly placed in a homeologous chromosome explained most of these conflicts. 156 SNPs (1.0% of the 15,497 SNPs) that mapped to genomic DNA unassigned to a pseudo-chromosome position mapped to linkage groups, thus revealing the locations of these sequences in the wheat genome (Table S5). For example, three SNPs were within 245,898 bp on an unmapped sequence contig, and the three mapped close to 154 cM on linkage group 4A (Figure S2). To identify QTL, multiple QTL mapping (MQM) was performed using MapQTL version 5 (Van Ooijen 2009). Additional information is in Supplemental Materials and Methods.

### Data availability

The analyses and data necessary for confirming this manuscript’s conclusions are publicly available. Data analysis protocols and code are provided in Supplemental Materials and Methods. Trait data are in Table S1. SNP calls across the DH population are in Table S4. RNASeq samples to identify Red Fife and Stettler SNPs and to calculate genes’ transcript abundances are available from NCBI (Bioproject ID PRJNA625320). Further information is available from the corresponding author if required. Supplemental material available at figshare: https://doi.org/10.25387/g3.12542930.

## Results

### With the exception of a semi-dwarfing gene, pleiotropic genes selected during wheat breeding favorably affected different target traits

Heading date, lodging susceptibility, plant height, protein content, spike length, thousand grain weight, and yield are important traits for wheat production and use, and varieties are selected based on these attributes. All traits significantly varied among the DH population generated from Red Fife and Stettler (Table 1). With the exception of plant height, traits’ genotypic distributions were close to normally distributed indicating polygenic effects (Figure S3). The plant height distribution was continuous but bimodal (Figure S3), indicating the effects of the major *Rht-B1* allele (Peng *et al.* 1999) and alleles from other, smaller effect loci (Zanke *et al.* 2014).

**Table 1:** Trait values, ranges, and heritabilities in the Red Fife and Stettler doubled haploid population

		Parent			DH population			
	Abb.*^a^*	RF	S	RF *vs.* S*^b^*	Mean	Range (Min-Max)	P-value	*h^2^_b_*
Heading	Hdg	58	56	ns	56.6	52-64	0.001	0.34
Grain protein content	Gpc	15	17	**	16	14-20	<0.001	0.45
Grain yield	Yld	132	310	**	172	44-363	0.001	0.37
Lodging susceptibility	Ldg	2.5	0	ns	10.7	0-100	<0.001	0.29
Plant height	Pht	89	72	**	79	55-105	0.005	0.83
Spike length	Spl	10	7.9	**	8.5	5.5-11	<0.001	0.63
Thousand grain weight	Tgw	26.3	25.6	ns	24.21	15.2-33.9	<0.001	0.71

a Units for traits. Heading (days after planting); Grain protein content (%); Grain yield (g.m^-2^); Lodging susceptibility (%); Plant height (cm); Spike length (cm); and Thousand grain weight (g).

b ns, *, and ** indicate non-significant and significant differences between parental trait values at 0.05 and 0.01 critical values, respectively.

**Figure 3:**
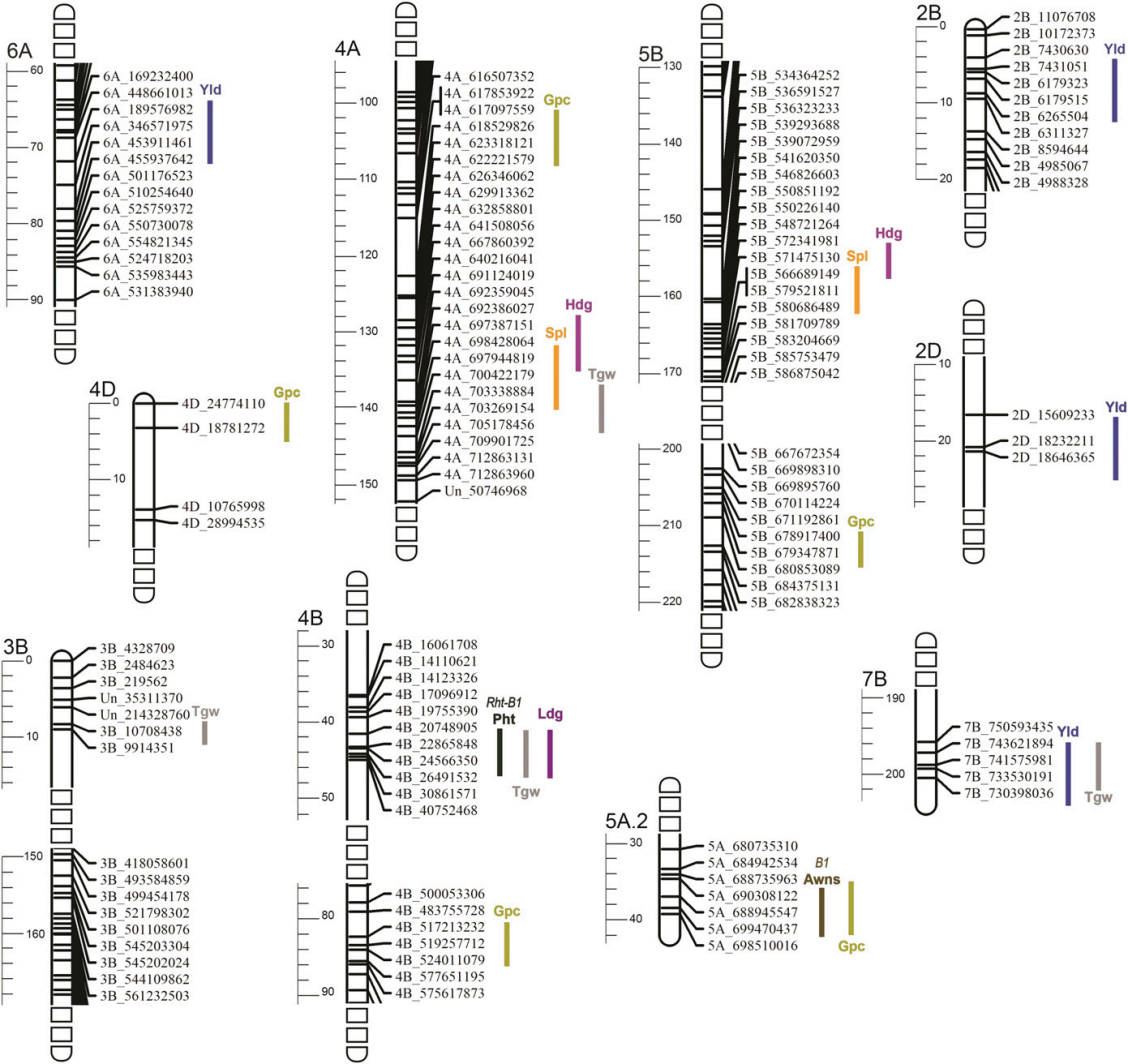
Linkage map intervals with QTL detected from the Red Fife x Stettler population. The cM distances are given on the axis on the left of each linkage group. Colored lines refer to QTL positions on the genetic map. Trait abbreviations and units are given in Table 1.

Stettler had similar thousand grain weight as Red Fife (Table 1), and notably higher yields and grain protein content. Positive selection appeared to favor loci with favorable pleiotropic effects (Table 2). Thousand grain weight positively correlated with yield (r = 0.53) and grain protein content (r = 0.44); and grain protein content and yield were positively correlated (r = 0.24). These traits also nominally, negatively correlated with spike length for which low values are favorable. QTL mapping identified two loci that contributed to this positive pleiotropy. A Stettler allele at a QTL on 7B contributed to yield and thousand grain weight, and a Stettler allele at a QTL on 4A reduced spikelet length and enhanced thousand grain weight (Figure 3, Table 3). In addition to effects of pleiotropic QTL, Stettler tended to have favorable alleles for nine QTL that were not pleiotropic. These included 5 grain protein content (3 with favorable Stettler alleles/ 2 with Red Fife favorable alleles), 3 grain yield QTL (2 Stettler/ 1 Red Fife), and 1 thousand grain weight QTL (1 Stettler/ 0 Red Fife) (Table 3).

**Table 2:** Pearson correlation coefficients between traits in the Red Fife x Stettler doubled haploid population

	Ldg*^a^*	Hdg	Pht	Spl	Tgw	Yld
Ldg	—					
Hdg	−0.15	—				
Pht	0.30	−0.25	—			
Spl	0.12	0.34	0.20	—		
Tgw	−0.13	−0.25	0.40	−0.13	—	
Yld	−0.20	−0.11	0.16	−0.14	0.53	—
Gpc	−0.09	−0.17	0.28	−0.15	0.44	0.24

a See Table 1 for trait key. Correlations’ absolute values >0.30, between 0.20 and 0.30, and between 0.15 and 0.20 are highlighted in dark grey, grey, and light grey respectively. All highlighted correlations are significant at *P* < 0.05.

**Table 3:** Locations and effect sizes of QTL

QTL	Abb.*^a^*	Chr	Conf. lnt (cM)	Left marker	Right marker	R^2^	Add. Effect*^b^*
QAwn.UG-5A.2 (*B1*)	Awn	chr5A	36-42	chr5A_698510016	chr5A_690308122	97.5	−0.50
QHdg.UG-4A	Hdg	chr4A	128-135	chr4A_632858801	chr4A_667860392	10.2	0.76
QHdg.UG-5B	Hdg	chr5B	153-157	chr5B_566689149	chr5B_572341981	8.7	−0.70
QYld.UG-2B	Yld	chr2B	4-12	chr2B_6179515	chr2B_6311327	16	−25.00
QYld.UG-2D	Yld	chr2D	17-25	chr2D_18232211	chr2D_28965350	12.6	−22.32
QYld.UG-6A	Yld	chr6A	64-72	chr6A_169232400	chr6A_189576982	11	20.66
QYld.UG-7B	Yld	chr7B	196-204	chr7B_733530191	chr7B_743621894	14.4	−23.64
QPht.UG-4B (*Rht-B1*)	Pht	chr4B	41-47	chr4B_26491532	chr4B_40752468	51.6	8.02
QTgw.UG-3B	Tgw	chr3B	5-11	chr3B_9914351	chrUn_214328760	12	−1.28
QTgw.UG-4A	Tgw	chr4A	137-143	chr4A_697387151	chr4A_692359045	9.5	−1.12
QTgw.UG-4B	Tgw	chr4B	41-47	chr4B_26491532	chr4B_40752468	14.7	1.41
QTgw.UG-7B	Tgw	chr7B	196-202	chr7A_700676957	chr7B_733530191	14.1	−1.39
QLdg.UG-4B	Ldg	chr4B	41-47	chr4B_26491532	chr4B_40752468	26	4.80
QGpc.UG-4A	Gpc	chr4A	101-108	chr4A_610493719	chr4A_606805750	11.9	−0.36
QGpc.UG-4B	Gpc	chr4B	81-86	chr4B_577651195	chr4B_519257712	9.7	0.33
QGpc.UG-4D	Gpc	chr4D	0-5	chr4D_24774110	chr4D_18781272	5.1	0.24
QGpc.UG-5A.2	Gpc	chr5A	35-42	chr5A_688945547	chr5A_698510016	6.2	−0.26
QGpc.UG-5B	Gpc	chr5B	211-215	chr5B_684375131	chr5B_684494988	6.9	−0.28
QSpl.UG-4A	Spl	chr4A	132-140	chr4A_640216041	chr4A_692359045	19.1	0.38
QSpl.UG-5B	Spl	chr5B	156-162	chr5B_566689149	chr5B_580686489	15.9	−0.35

a QTL trait abbreviations are given in Table 1.

b A positive effect indicates that the Red Fife allele positively contributes to the trait value.

Plant height was the exception to Stettler alleles’ favorable pleiotropy. Favorable (short) plant height values were associated with unfavorable values for other traits. Shorter plants had significantly lower yields (r = 0.16), grain protein content (r = 0.28), and thousand grain weight (r = 0.40) than taller plants (Table 2). Plant height was driven by the large effect *Rht-B1* locus. *Rht-B1* explained 52% of variation for height (Table 3) and contributed to reduced thousand grain weight (Table 3, Figure 3). Lines homozygous for the Stettler *Rht-B1* allele had a thousand grain weight 2.8g lower than lines homozygous for the Red Fife allele (Table 3).

Despite the unfavorable genetic correlations between plant height and yield, grain protein content, and thousand grain weight, Stettler’s yield and grain protein content notably exceeded that of Red Fife, and its thousand grain weight did not significantly differ (Table 1). In contrast, the height difference between Red Fife and Stettler (16 cm; Table 1) was almost identical to the difference between *Rht-B1* homozygous classes (Table 3, Figure S3). We detected three alleles that compensated for *Rht-B1*’s unfavorable effects. Stettler alleles at 3B, 4A, and 7B QTL all increased thousand grain weight (Figure 3, Table 3). The 3B and 4A QTL may correspond to previously identified loci (Quraishi *et al.* 2017), although parents of the QTL mapping populations in which these alleles were identified (Börner *et al.* 2002; Huang *et al.* 2004) were not in Stettler’s pedigree (McCallum and DePauw 2008).

Heading date was under stabilizing selection because of its importance to adaptation and did not significantly differ between Red Fife and Stettler. Nonetheless, the continuous distribution of heading dates in the DH population showed that genetic basis of heading date differed between Red Fife and Stettler (Figure S3). Heading date alleles affected spike length (r = 0.34) (Table 2). A Stettler allele at a 5B QTL increased heading date and spike length, and the Stettler allele at a 4A QTL, decreased heading date and spike length (Figure 3, Table 3). Meristem maintenance in different developmental contexts is developmentally linked (Yan *et al.* 2011) and may explain the genetic correlation.

### SNPs in closely related chromosomal regions occurred at conserved sites and appeared unaffected by selection

Next, we investigated the attributes of mutations that likely occurred within the timeframe of modern plant breeding. Related chromosomal regions between Red Fife and Stettler accounted for 17% of the genome, contained 25% of all genes, and had 60 coding region SNPs. The transition frequencies in related regions was high (70.0%) and similar to the frequency in unrelated regions (77.0%; Fisher’s Exact Test, *P* > 0.05; Table 4). These frequencies were similar to those observed between other wheat alleles (Lai *et al.* 2015). The Red Fife seed used in this study was from self-pollination of the ancestral cultivar, so mutations accumulated in both Red Fife and Stettler lineages. The Chinese Spring nucleotide can define recent mutations’ ancestral states. The frequency of G:C to A:T transitions among recent mutations was high in both in the current Red Fife and Stettler lineages (27/60; 45%) (Table 4). G:C to A:T transitions account for over 40% of the mutations that arose in *Arabidopsis thaliana* identity by descent regions (Exposito-Alonso *et al.* 2018) likely because of the high rate of mutation from methylated cytosine to uracil (Coulondre *et al.* 1978).

**Table 4:** Numbers and frequencies of nucleotide transitions and transversions between Red Fife and Stettler within related and unrelated chromosomal regions

	Transition		Transversion				
	G:C→A:T	A:T→G:C	A:T→C:G	G:C→T:A	A:T→T:A	G:C→C:G	Ts:Tv
Unrelated*^a^*		14,783		3,416	1,086	2,675	2
		(0.77)		(0.18)	(0.05)	(0.12)	
Related*^a^*		42		10	2	6	2.3
		(0.70)		(0.17)	(0.03)	(0.10)	
RF_A_->RF_C_*^b^*	16	7	2	4	1	3	2.3
RF_A_->S*^b^*	11	8	1	3	1	3	2.4

a Nucleotide differences counted when the Red Fife allele is compared against the Stettler allele and visa-versa.

b The direction of substitutions between the ancestral Red Fife (RF_A_) and the current Red Fife (RF_C_) and Stettler (S) can be inferred using the Chinese Spring allele as the outgroup. See Figure 2 substitution types 3 and 4.

Despite similar transition frequencies between putatively new and old polymorphisms, the former had much more severe effects on proteins than did the latter. Predicted high and moderate effect coding SNPs were over-represented in related chromosomal regions relative to unrelated regions (Fisher’s Exact Test, *P* < 0.001). Among the 60 SNPs, 1 SNP (1.7%) had a high effect, 41 (68.3%) had moderate effects, and 18 (30.0%) had low effects (Table 5). By comparison, among the 13,133 SNPs outside of related regions, 79 (0.6%) had high effects, 5,975 (45.5%) had moderate effects, and 7,079 (53.9%) had low effects (Table 5). The distribution of related SNP effects was almost identical to the distribution obtained when randomly assigning Red Fife/ Stettler polymorphisms to expressed genes. Among 99,993 simulated SNPs, 3,056 (3.1%) had high effects; 68,514 (68.5%) had moderate effects; and 28,423 (28.4%) had low effects (Table 5) (Chi-squared Test; *P* = 0.81). We had expected mutations within evolutionarily conserved sequences to have been removed in both the current Red Fife and Stettler lineages, and for the removal to be highest in the Stettler lineage due to stronger selection. Missense mutations accumulated in similar numbers in the current Red Fife and Stettler lineages. Of the 60 SNPs in related regions, 33 occurred within the current Red Fife lineage (1 high effect; 21 moderate; and 11 low). These mutations had significantly larger effects than SNPs in unrelated regions (Fisher’s Exact Test; *P* = 0.02) and did not significantly differ from random mutations (Fisher’s Exact Test; *P* = 0.64). Similarly, twenty-seven mutations occurred within the Stettler lineage (0 high; 20 moderate; and 7 low). These mutations also had significantly larger effects than SNPs in unrelated regions (Fisher’s Exact Test; *P* = 0.02) and also did not significantly differ from random mutations (Fisher’s Exact Test; *P* = 0.93) (Table 5). Breeders’ selections appear to have had no effects on the fates of novel mutations.

**Table 5:** Numbers and frequencies of SNPs between Red Fife and Stettler with high, low, and moderate effects on protein function in both related and unrelated chromosomal regions

SNP Type	HIGH	LOW	MODERATE	Total
Unrelated*^a^*	74	7,082	5,977	13,133
	(0.01)	(53.93)	(45.51)	
Random*^b^*	3,056	28,423	68,514	99,993
	(3.05)	(28.42)	(68.52)	
Related*^a^*	1	18	41	60
	(1.66)	(30.00)	(68.33)	
RF_new*^c^*	1	11	21	33
	(3.03)	(33.33)	(63.64)	
S_new*^c^*	0	7	20	27
	(0.00)	(25.93)	(74.07)	

a Unrelated SNPs between Red Fife and Stettler are the SNPs outside of those found in closely related, putatively identical by descent regions.

b Random SNPs were generated in silico. See text for details.

c RF_new indicates that the Red Fife nucleotide in a related region differed from both Chinese Spring and Stettler. S_new indicates that the Stettler nucleotide differed from both Chinese Spring and Red Fife.

Recent polymorphisms also occurred at similar frequencies in conserved genes and less conserved genes. Although purifying selection on duplicated genes is often weaker than on non-duplicated genes (Gu *et al.* 2003), Appels *et al.* (Appels *et al.* 2018) reported that within the wheat genome, genes that had three copies were more likely to have orthologs in other species than genes that had fewer than three copies. Consistent with the concept that triplicated genes are highly conserved, genes in three or more homeologous copies, termed unique triplet and non-unique triplet genes, respectively, had significantly fewer SNPs between Red Fife and Stettler than did non-triplicated genes. Medians were 0.82 SNPs per kb for non-triplicate genes, 0.51 SNPs per kb for non-unique triplicates, and 0.56 for unique triplicates (Table 6, *P* < 0.001). In unrelated genomic regions, non-triplicated genes accumulated more SNPs relative to unique and non-unique triplicated genes (1.67 *vs.* 0.92 and 1.04 *P* < 0.0001; Table 6). In contrast, related genomic regions had similar numbers of new mutations among the three types of genes (Table 6).

**Table 6:** Frequencies of SNPs among triplicated and non-triplicated genes within related and unrelated chromosomal regions

	SNPs per kb		
	non_Triplet	nonUnique_Triplet	unique_Triplet
Overall*^a^*	0.82a	0.51b	0.56b
Related*^a^*	0.54a	0.42a	0.53a
Unrelated*^a^*	1.67a	0.92b	1.04b

a In each row, numbers with different letters were significantly different according to Tukey’s multiple comparison test.

Finally, the sequences of highly expressed genes can be more highly conserved over evolutionary time than the sequences of genes expressed at low levels (Liao and Zhang 2006). Transcript levels of genes with new mutations (median normalized read count, 136.4) were not significantly higher than levels of unrelated genes with SNPs (134.8; Wilcoxon rank sum test, *P* = 0.50).

## Discussion

### Wheat breeding did not remove nonsynonymous mutations and mutations in conserved genes

One objective of this work was to determine if putatively deleterious mutations accumulated during plant breeding or were eliminated by breeders’ selections. New polymorphisms occurred at high frequency within conserved sequences. The proportion of nucleotide differences that were nonsynonymous or nonsense, 0.70, was much higher than the proportion within unrelated regions 0.46 (Table 5). New polymorphisms also accumulated within conserved, triplicated genes at a frequency similar to less conserved, non-triplicated genes (Table 6). Polymorphisms’ effects on amino acid sequences were very similar to those arising from chance alone (Table 5) and very similar to the ratios observed within clonal *Arabidopsis thaliana* populations propagated with no selection. Ossowski (Ossowski *et al.* 2010) found 11 nonsynonymous mutations and 4 synonymous mutations in mutation accumulation lines propagated in a uniform environment.

Three explanations can explain why new polymorphisms occur at high frequencies within normally conserved sequences despite very strong selection for favorable genotypes. First, these mutations may be favorable and were positively selected. Mutations can generate valuable trait diversity *e.g.*, (Rutter *et al.* 2010; Jiang *et al.* 2011; Exposito-Alonso *et al.* 2018), and selection appears to maintain variants that are locally adaptive but deleterious elsewhere (Fournier-Level *et al.* 2011). Nonetheless, while Shaw *et al.* (Shaw *et al.* 2000) found mutational effects on Arabidopsis reproductive traits were symmetrically distributed, favorable new mutations are rare (Gossmann *et al.* 2010), and in almost all coding sequence comparisons, the frequency of nonsynonymous differences is low relative to synonymous differences because selection has eliminated the former (Nielsen and Slatkin 2013). Thus, we expect that selection favored few if any new mutations. Consistent with this interpretation, no QTL influencing key traits mapped to closely related chromosomal regions between Red Fife and Stettler (Table 3). Second, these variants may have had notable, negative effects on target traits but nonetheless accumulated. This explanation is unlikely because of the plant breeding and cultivar approval processes. As noted (Figure 1A), breeders evaluate target traits in 1000s of homozygous or highly homozygous lines replicated over many locations and years and select a small number for commercial release. Genotypes with low target trait values do not become cultivars. As with other crops, wheat yields have steadily increased due to genetic improvement (Mackay *et al.* 2011; Laidig *et al.* 2017). Selection acts on the net effect of favorable and unfavorable mutations. Mutations with notable negative effects on target traits could arise in a line with many positive alleles, but as noted, this also seems unlikely. Since breeding populations typically arise from crosses between two homozygous parents, a line in which a deleterious mutation arises would need to out-perform genetically similar full-sibling derived lines without the deleterious mutation. The third and our favored explanation is that nonsynonymous mutations that accumulated during wheat breeding likely have no effects on selected traits or effects that are too small to have an impact on selection decisions. The number of genes whose conserved functions are not required to advance breeders’ target traits is likely high. In an absence of purifying selection, only lethal or extremely deleterious alleles will not be represented in a population (Barrick and Lenski 2013). Consistent with this idea, the effects of nucleotide changes that occurred in the Stettler lineage that was under strong selection for favorable traits were similar to the effects of changes that occurred in the Red Fife seed maintenance lineage that had no intentional selection (Table 5). Nucleotide differences at conserved sequences have been termed putatively deleterious for elite cultivars, and their removal has been a suggested goal of crop improvement (Morrell *et al.* 2012). These results suggest selecting against these alleles would have little effect on plant performance. In addition, several reports have reported a domestication cost, whereby the frequency of deleterious alleles in domesticated populations has increased relative to wild populations (Lu *et al.* 2006; Mezmouk and Ross-Ibarra 2014; Renaut and Rieseberg 2015; Liu *et al.* 2017). Factors that could increase the frequency of deleterious mutations- genetic drift *e.g.*, (Makino *et al.* 2018); expanding populations of domesticates *e.g.*, (Peischl *et al.* 2013); and linkage of deleterious alleles with favorable alleles *e.g.*, (Hill and Robertson 1966)- apply to domestication. Nonetheless, a notable proportion of these putative deleterious variants may also have had no negative effects on domesticated crops.

This study identified and characterized putative, recent mutations in young plants’ expressed genes. While a plant’s healthy, early development is a prerequisite for high grain yields at the end of its life-cycle, missense mutations in genes expressed in tissues more closely related to breeders’ target traits such as developing inflorescences, florets, or seeds, could more likely have deleterious effects and be eliminated by selection. Genes with tissue specific expression evolve more quickly at the protein level than genes expressed across tissues (Movahedi *et al.* 2011). New mutations within genes expressed in multiple tissues may be most effectively removed in breeding programs.

Unlike recent polymorphisms, the frequency of older variants indicates past, purifying selection. The primary reason is likely because wheat like other crops was domesticated only within the last 1,000s of years (Meyer and Purugganan 2013), and cultivars’ alleles derive from wild standing variation. Selection in a wild population would likely act on far more alleles than Canadian breeding programs which target specific traits in a limited set of environmental conditions and genetic backgrounds. Purifying selection would also likely affect more alleles in wild populations as these alleles are utilized more broadly than within plant breeding populations. In plant breeding, as in animal breeding (Parker *et al.* 2017) and animal conservation (Araki *et al.* 2007), the traits (and thus alleles) favorable in one context may not be favorable in another.

### Loci selected during breeding were favorably pleiotropic, and compensatory loci counteracted Rht-B1b’s undesirable effects

Our genetic analyses suggested that wheat breeding targeted loci with favorable, pleiotropic effects. With the exception of plant height, traits were correlated such that DH genotypes with a favorable value for one trait were expected to have a favorable value for another trait. Yield correlated with thousand grain weight; yield correlated with grain protein content; and thousand grain weight correlated with grain protein content (Table 2). These three traits were also nominally, favorably correlated with spike length (Table 2). While genetic correlations could be trivial if traits are developmentally linked such that a change in one trait results in a change in another, these examined traits are often uncorrelated. For example, although yield is a function of seed size and seed weight, yield and seed size may be experimentally separated. In the long-term evolution of a barley population, increases in grain yield were due to increases in the number of small kernels on compact spikes (Allard 1988). Protein content and yield are widely, negatively correlated in cereals (Simmonds 1995) perhaps because of competition between carbon and nitrogen storage molecules for energy (Munier-Jolain and Salon 2005). Thus, the genetic correlation between yield and protein suggests that Stettler alleles enhance overall resource availability *e.g.*, (van Noordwijk and de Jong 1986) thereby allocating more energy to both carbon and nitrogen storage molecules. Notably, genetic values were only in part due to correlations, and favorable Stettler alleles accumulated at QTL that affected only one trait (Table 3). Similarly, in rice breeding, eQTL alleles with consistent regulatory effects on gene expression accumulated in a single lineage (House *et al.* 2014).

The favorable trait correlations were likely due to pleiotropic loci that accumulated in the Stettler lineage and had small, favorable effects on traits. We identified only two QTL- a Stettler 4A allele that decreased spike length and increased thousand grain weight, and a Stettler 7B allele that increased yield and thousand grain weight (Table 3)- that contributed to trait correlations. Red Fife, Stettler, and lines utilized during Stettler breeding (Figure 1B) are high quality cultivars that do not typically segregate for large effect loci that affect important quantitative traits. In contrast, Allard (1988) identified several large effect loci that contributed to multiple traits in a barley long term evolution population. This population was derived from 28 highly diverse barley varieties, many of which were poorly adapted to the trial location. Identifying those genes with favorable pleiotropic effects within our population would be an important contribution to understanding the processes that contribute to crop improvement. QTL span long chromosomal regions because linkage disequilibrium is high within our mapping population. CRISPR technology could evaluate SNPs within genes spanned by QTL. Potential SNPs are provided in Table S7. For example, a C/T polymorphism distinguishes the *Rht-B1a* wild type from the *Rht-B1b* dwarfing allele (Peng *et al.* 1999). This site was detected in our alignments, and SnpEff labeled the nucleotide as having high effect on the predicted protein.

Stettler’s favorable pleiotropic alleles will unlikely be favorable in all breeding contexts. Different combinations of life history traits are favored in different environments. For example, an allele that causes early heading dates and a short spike lengths (Table 2, Table 3) may be favorable for Canadian wheat because of the short growing season. However, this association would not be favorable if breeders desire later flowering and short spikes. The effects of pleiotropic genes advantageous to Stettler improvement could also differ across genetic backgrounds and across different environments. Although plant breeding and domestication targets stable crop performance across environments (Doust *et al.* 2014; Qian *et al.* 2017), loci may not reveal the same pleiotropic effects in different genetic backgrounds or in different locations (Pavličev and Cheverud 2015).

Unlike other traits, favorable, short plant heights co-occurred with unfavorable yields, grain protein content, and thousand grain weights within the DH population (Table 2). Nonetheless, Stettler, despite being semi-dwarf, had higher yield and protein content than Red Fife and a similar grain weight (Table 1). The *Rht-B1*b allele generated the desired height for Stettler but reduced thousand grain weight, and Stettler alleles at three QTL on 3B, 4A, and 7B increased grain weight to compensate (Table 1, Table 3). Remarkably, Sherman *et al.* (Sherman *et al.* 2014) reported that *Rht-D1*, a homeologous, green revolution dwarfing gene, had unfavorable grain weight effects that required compensation in American breeding. Thus, although we anticipated negatively pleiotropic genes could be utilized in wheat breeding populations, in over a century of wheat improvement efforts, we detected only one large effect locus with favorable and unfavorable effects. Breeders seek for genotypes to occupy the best possible position in multi-variate trait space, and selection for negatively pleiotropic genes would impede genetic progress *e.g.*, (Mitchell-Olds 1996).

It would be intriguing to achieve semi-dwarfism without the *Rht-B1b* allele. Perhaps other *Rht-B1* alleles would not have deleterious effects. For example, Ookawa *et al.* 2010 (Ookawa *et al.* 2010) identified a gain of function mutant of the rice APO allele, SCM2, that enhanced culm strength and increased spikelet number but did not have the negative effects of other APO1 alleles. Alternatively, many height modifying genes segregate within this and other wheat germplasm (Figure S3) (Zanke *et al.* 2014). Selection for alleles without unfavorable effects may be able to change plant height.

## Conclusion

This article reports two key findings. First, mutations that likely occurred in the course of plant breeding appeared unaffected by purifying selection. Second, although plant breeders have utilized alleles with favorable effects on one quantitative trait and negative effects on others by compensating for the latter, *Rht* semi-dwarfing genes are the only examples of this in our analyses. Wheat improvement was due to small to moderate effect alleles with positively pleiotropic effects or no pleiotropic effects. Our genetic and molecular results arise from the examination of a single lineage, so strong conclusions are speculative. Nonetheless, we expect that many nucleotide changes that have occurred at conserved sites during plant improvement have had little effect on desirable traits. As sequences of other ancestors within plant pedigrees become available, the patterns and consequences of novel mutations will be made clear. Similarly, we expect that in general selection for important quantitative traits preferentially acts upon alleles that are favorable for one or multiple traits, and selection for large effect single genes like *Rht-B1* that require compensation is rare. Future crosses between ancestral and modern cultivars may test this hypothesis.
